# Boosting Deep Learning for Interpretable Brain MRI Lesion Detection through the Integration of Radiology Report Information

**DOI:** 10.1148/ryai.230520

**Published:** 2024-10-09

**Authors:** Lisong Dai, Jiayu Lei, Fenglong Ma, Zheng Sun, Haiyan Du, Houwang Zhang, Jingxuan Jiang, Jianyong Wei, Dan Wang, Guang Tan, Xinyu Song, Jinyu Zhu, Qianqian Zhao, Songtao Ai, Ai Shang, Zhaohui Li, Ya Zhang, Yuehua Li

**Affiliations:** From the Institute of Diagnostic and Interventional Radiology (L.D., Z.S., H.D., J.J., D.W., G.T., X.S., J.Z., Q.Z., Y.L.) and Clinical Research Center (J.W.), Shanghai Sixth People’s Hospital Affiliated to Shanghai Jiao Tong University School of Medicine, 600 Yishan Road, Shanghai 200000, China; Shanghai AI Laboratory, Shanghai, China (J.L., Y.Z.); School of Computer Science and Technology, University of Science and Technology of China, Anhui, China (J.L.); The Pennsylvania State University College of Information Sciences and Technology, University Park, Pa (F.M.); Department of Electrical Engineering, City University of Hong Kong, Hong Kong, China (H.Z.); Department of Radiology, Affiliated Hospital of Nantong University, Nantong, China (J.J.); Department of Radiology, Shanghai Ninth People’s Hospital Affiliated to Shanghai Jiao Tong University School of Medicine, Shanghai, China (S.A.); Department of Radiology, Shanghai Public Health Clinical Center, Shanghai, China (A.S.); Department of Radiology, Wuhan Hankou Hospital, Wuhan, China (Z.L.); and Cooperative Medianet Innovation Center, Shanghai Jiao Tong University, Shanghai, China (Y.Z.).

**Keywords:** Deep Learning, Computer-aided Diagnosis, Knowledge-driven Model, Radiology Report, Brain MRI

## Abstract

**Purpose:**

To guide the attention of a deep learning (DL) model toward MRI characteristics of brain lesions by incorporating radiology report–derived textual features to achieve interpretable lesion detection.

**Materials and Methods:**

In this retrospective study, 35 282 brain MRI scans (January 2018 to June 2023) and corresponding radiology reports from center 1 were used for training, validation, and internal testing. A total of 2655 brain MRI scans (January 2022 to December 2022) from centers 2–5 were reserved for external testing. Textual features were extracted from radiology reports to guide a DL model (ReportGuidedNet) focusing on lesion characteristics. Another DL model (PlainNet) without textual features was developed for comparative analysis. Both models identified 15 conditions, including 14 diseases and normal brains. Performance of each model was assessed by calculating macro-averaged area under the receiver operating characteristic curve (ma-AUC) and micro-averaged AUC (mi-AUC). Attention maps, which visualized model attention, were assessed with a five-point Likert scale.

**Results:**

ReportGuidedNet outperformed PlainNet for all diagnoses on both internal (ma-AUC, 0.93 [95% CI: 0.91, 0.95] vs 0.85 [95% CI: 0.81, 0.88]; mi-AUC, 0.93 [95% CI: 0.90, 0.95] vs 0.89 [95% CI: 0.83, 0.92]) and external (ma-AUC, 0.91 [95% CI: 0.88, 0.93] vs 0.75 [95% CI: 0.72, 0.79]; mi-AUC, 0.90 [95% CI: 0.87, 0.92] vs 0.76 [95% CI: 0.72, 0.80]) testing sets. The performance difference between internal and external testing sets was smaller for ReportGuidedNet than for PlainNet (Δma-AUC, 0.03 vs 0.10; Δmi-AUC, 0.02 vs 0.13). The Likert scale score of ReportGuidedNet was higher than that of PlainNet (mean ± SD: 2.50 ± 1.09 vs 1.32 ± 1.20; *P* < .001).

**Conclusion:**

The integration of radiology report textual features improved the ability of the DL model to detect brain lesions, thereby enhancing interpretability and generalizability.

**Keywords:** Deep Learning, Computer-aided Diagnosis, Knowledge-driven Model, Radiology Report, Brain MRI

*Supplemental material is available for this article.*

Published under a CC BY 4.0 license.

**Summary**

A deep learning model incorporating radiology report–derived textual features outperformed a basic deep learning model trained without textual features in identifying 14 brain diseases and normal brain on MR images while providing interpretable attention maps.

**Key Points**

■ A deep learning model (ReportGuidedNet), developed without manual image annotation but with incorporation of radiology reports, outperformed the basic model (PlainNet) in identifying 14 brain diseases and normal brains, as evidenced by higher macro-averaged area under the receiver operating characteristic curve (AUC) (0.93 [95% CI: 0.91, 0.95] vs 0.85 [95% CI: 0.81, 0.88]) and micro-averaged AUC (0.93 [95% CI: 0.90, 0.95] vs 0.89 [95% CI: 0.83, 0.92]) on the internal test set.
■ ReportGuidedNet produced more accurate and interpretable lesion localization attention maps than PlainNet, as evidenced by a higher Likert scale score (mean ± SD, 2.50 ± 1.09 vs 1.32 ± 1.20; *P* < .001).
■ ReportGuidedNet showed less performance difference than PlainNet on internal and external testing sets (Δma-AUC, 0.03 vs 0.10; Δmi-AUC, 0.02 vs 0.13).

## Introduction

Brain MRI is essential for diagnosing and evaluating brain diseases. Brain MRI lesions are highly heterogeneous, resulting in error-prone subjective analysis (1). Deep learning (DL) models have been developed to assist radiologists in the clinic. However, their training can be hindered by inadequate expert annotations and limited domain knowledge guidance, leading to fluctuating diagnostic accuracy and insufficient interpretability (2–4).

To direct the attention of a DL model to capture lesion features, a commonly used strategy involves manual segmentation of image regions (5–7). However, this approach presents limitations. First, certain brain lesions, because of their indistinct boundaries, irregular shapes, or high quantity, necessitate costly, laborious, and inconsistent labeling processes. Furthermore, some lesions cause morphologic and signal intensity changes in adjacent normal brain tissue, such as mass effect and vasogenic edema, which are critical for differential diagnosis. Merely segmenting lesions falls short in quantifying these alterations. Finally, certain diseases exhibit global brain changes instead of focal alterations. An improved image annotation method that addresses these limitations is therefore needed.

Radiology reports are meticulously crafted and reflect the train of thought and reasoning of radiology experts during interpretation of medical images; they provide detailed descriptions of brain lesions characteristics and anatomic alterations in extensive real-world clinical cases and are organized in a standardized manner. Radiology reports are naturally and seamlessly generated during the clinical diagnostic process, incurring no supplementary production costs. Consequently, they can serve as a high-accuracy, low-cost source of weak annotations for MR images.

Previous studies (8–11) have accomplished multimodal image-text pretraining through the application of contrastive learning on chest radiographs and corresponding radiology reports. This involved the use of vision and text encoders or pretrained models customized for radiology-specific contexts. In contrast to radiographs, MR images are characterized by multiple sequences, which offer better tissue contrast and can help discern lesions with complex components, leading to more intricate and rich textual features in their reports.

An effective diagnostic aid model should provide accurate and stable diagnostic results, offering interpretable indicators to substantiate its predictions as references for radiologists. To achieve this in a cost-effective manner, we propose an approach that uses data derived from radiology reports to enhance the attention of a DL model toward lesion characteristics. The current study aimed to facilitate lesion detection at brain MRI using this approach, while generating interpretable attention maps without the need for manual image segmentation during model training.

## Materials and Methods

### Study Design

This multicenter retrospective study was approved by the local ethics committee (approval no. 2023-KY-082 [K]), with waiver of informed consent, and involved five tertiary hospitals.

### Dataset Collection

**Figure 1:**
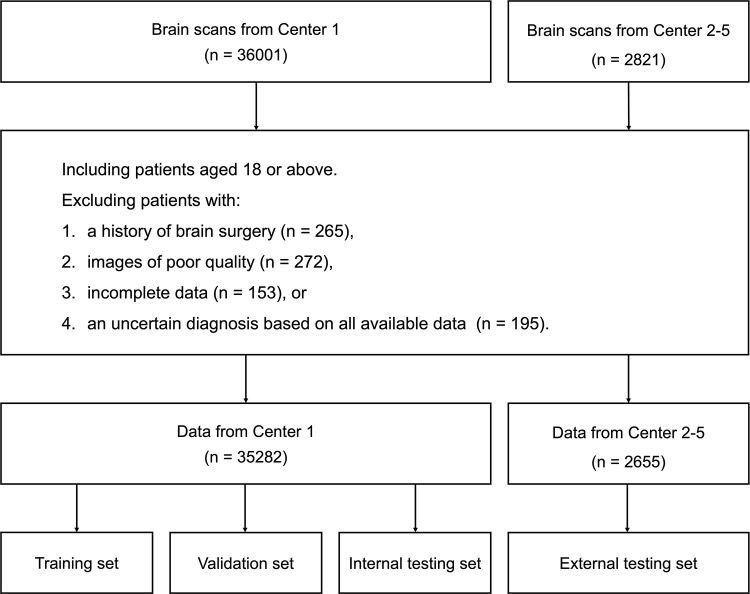
Flowchart of data screening.

For the training, validation, and internal testing datasets, we retrospectively gathered noncontrast brain MR images, along with the corresponding radiology reports and clinical data from center 1 (The Sixth People’s Hospital Affiliated to Shanghai Jiao Tong University School of Medicine), spanning from January 2018 to June 2023. For the external testing datasets, noncontrast brain MR images from centers 2–5 (center 2: Ninth People’s Hospital Affiliated to Shanghai Jiao Tong University School of Medicine; center 3: Fudan University Shanghai Public Health Center; center 4: Affiliated Hospital of Nantong University; center 5: Wuhan Hankou Hospital) acquired from January 2022 to December 2022 were used. To ensure the privacy and confidentiality of patient data, all radiology reports were de-identified, with personal identifiers removed and replaced with a unique identification number to match the corresponding MR images. All participating centers adhered to identical eligibility criteria by including adult (≥18 years) outpatients and inpatients who met the criteria for brain noncontrast MRI scans (Fig 1).

Exclusion criteria were as follows: *(a)* patients with history of brain surgery due to potential anatomic alterations; *(b)* scans with low image quality, defined through radiologist assessments as those in which diagnostic accuracy was compromised by artifacts or improper settings (this is in accordance with our clinical standard operating procedures and marked in the picture archiving and communications system software); *(c)* cases with incomplete data (unfinished examination or missing images, reports, or medical records due to system errors); and *(d)* cases with uncertain diagnosis, whereby a definitive diagnosis could not be determined after comprehensive discussion and review of all available medical data during the reference standard labeling process.

All included patients underwent the same noncontrast MRI sequences, consisting of T1-weighted imaging, T2-weighted imaging, T2 fluid-attenuated inversion recovery, diffusion-weighted imaging, and apparent diffusion coefficient mapping. In patients who underwent multiple brain imaging examinations during the study period, only the earliest scan was included. Image acquisition and image preprocessing details are presented in sections 1 and 2 of Appendix S1. The radiology reports were drafted by 68 junior radiologists and subsequently reviewed by 28 senior radiologists.

### Radiology Reports Feature Mining

We developed a natural language processing (NLP) algorithm to extract radiologic descriptions from radiology reports through the identification and matching of radiology terms. The ensuing structured radiology report comprised comprehensive information concerning lesion lateralization, location, size, morphologic features, signal characteristics, and perilesional changes across MRI sequences. To vectorize text features in the structured report, a knowledge-enhanced pretrained text encoder (8) was used, with careful exclusion of the impression section of the radiology report to prevent any potential label leakage. The process used for data mining of radiology reports is detailed in section 3 of Appendix S1.

### Model Development

To assess the effect of radiology report–derived data on the model, we developed a DL model, referred to as ReportGuidedNet, which incorporated radiology report–derived text features. For comparative analysis, we also established a vanilla DL model, PlainNet, which lacks these report-derived textual features.

**Figure 2:**
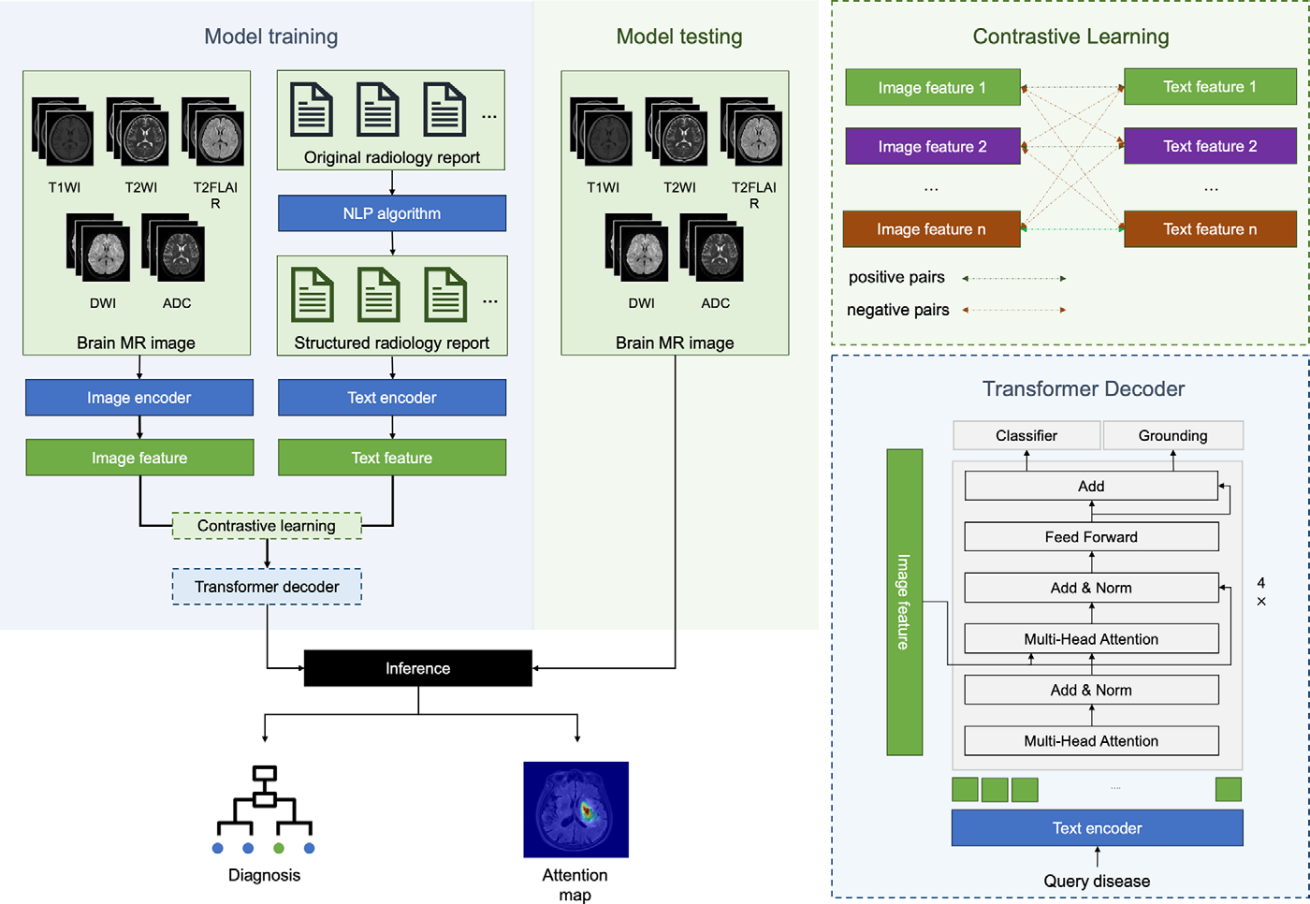
Diagram for the development of ReportGuidedNet. ADC = apparent diffusion coefficient, DWI = diffusion-weighted imaging, NLP = natural language processing, T1WI = T1-weighted imaging, T2FLAIR = T2 fluid-attenuated inversion recovery, T2WI = T2-weighted imaging.

The development of ReportGuidedNet adhered to the vision-language training paradigm, as shown in Figure 2. For each MRI sequence, we used a shared image encoder (12) to extract image features. These features are then concatenated and projected to a low-dimensional global image feature, yielding a comprehensive global image feature that incorporates information from all MRI sequences. Subsequently, a contrastive learning strategy was applied, enhancing the representation of MR images by leveraging structured textual features extracted from radiology reports for the integration of textual and image features. The enhanced image features and disease queries were input into a transformer decoder (13) to generate a multiclass diagnosis result. The objective of the model is to ascertain the presence of lesions, offering a multiclass diagnosis by categorizing the detected lesions according to the respective disease. Instances devoid of lesions are marked as normal brain. The model development is further described in section 4 of Appendix S1. The relevant code is publicly available at *https://github.com/LisongDai/ReportGuidedNet*.

In addition, to examine the necessity of structuring the reports, a model similar to ReportGuidedNet, termed UnstructuredReportGuidedNet, was formulated using the same training methods but integrated text features derived from original, unstructured radiology reports (section 6 of Appendix S1).

### Anomaly Localization

We incorporated the class activation mapping (14) technique and an attention module to generate three-dimensional attention maps. These maps visualized the specific image regions capturing the attention of the model, substantiating the model’s diagnostic decisions. These maps can provide radiologists with interpretable references and help identify crucial areas that may harbor disorders.

### Reference Standard Labeling

Reference standard labeling was performed by two neuroradiologists (X.S. and A.S., each with 5 years of experience) who divided the cases equally, with each neuroradiologist responsible for half of them, and independently reviewed all accessible medical data, including medical history and surgical, laboratory, pathology, and radiology records. In situations involving uncertainty, ambiguity, or disagreement regarding reference standard labeling, the two neuroradiologists convened to achieve consensus. Cases for which a definitive reference standard label could not be determined after comprehensive discussion and review of all available medical data were classified as “uncertain diagnosis” and excluded. Cases featuring lesions from various diseases were assigned multiple positive labels.

### Model Evaluation

The accuracy of the NLP algorithm was assessed by comparing the original radiology report with the structured radiology report (section 6 of Appendix S1). The anomaly detection by the attention map was evaluated using the pointing game’s precision and a five-point Likert scale (section 8 of Appendix S1). Patient-level accuracy was calculated as the ratio of patients correctly diagnosed with all their conditions without any errors or omissions to the total number of patients evaluated. Data size reduction and ablation experiments using various MRI sequences were performed to assess the influence of data size and composition on the model. The clinical utility of ReportGuidedNet was evaluated by comparing the diagnostic performance of unassisted radiologists with that of model-assisted radiologists in using a randomized crossover design with a 2-week washout period (section 9 of Appendix S1). Failure analysis was performed to evaluate the limitations of the ReportGuidedNet.

### Statistical Analysis

The Mann–Whitney *U* test was used for two independent non–normally distributed samples, and the Kruskal–Wallis test was used for more than two samples. For categorical variables, comparisons were made using the χ^2^ test or Fisher exact test. Model performance was assessed by calculating sensitivity, specificity, and accuracy, as well as by plotting receiver operating characteristic curves, computing the area under the receiver operating characteristic curve (AUC), and plotting calibration curves. Bootstrap resampling (10 000 replicates) was used to determine 95% CIs. The AUC was compared using the DeLong test. The overall performance of the model across all diseases was assessed using macro-averaged AUC (ma-AUC) and micro-averaged AUC (mi-AUC) (section 10 of Appendix S1). The ma-AUC and mi-AUC were compared using bootstrap sampling to calculate the 95% CI for the differences in AUC between the models. Spearman correlation analysis was used to evaluate the relationship between lesion size and attention map scores. A two-tailed *P* value less than .05 signified statistical significance. All statistical analyses were conducted using R software (version 4.3.1; R Project for Statistical Computing) and RStudio software (version 2023.06.2; R Project for Statistical Computing).

## Results

### Study Sample

A total of 37 973 brain MRI scans (mean ± SD age of patients: 54.7 years ± 19.0; 17 712 male and 20 261 female) were included in the study (Table 1). The dataset consisted of 94 385 lesions from center 1 and 6545 lesions from centers 2–5. These lesions encompassed 14 diseases: acute and subacute infarct, lacunar infarct, chronic infarct, cerebral hemorrhage, epidural and subdural hemorrhage, cerebral herniation, meningioma, acoustic neuroma, cavernoma, metastasis, glioma, hydrocephalus, cerebral atrophy, and white matter hyperintensities. In conjunction with the identification of a normal brain, our model achieved a total of 15 diagnoses. Patients’ age distribution and the prevalence of 14 diagnoses, excluding cerebral herniation, significantly differed between internal and external datasets. Detailed patient demographic characteristics are provided in section 1 of Appendix S1.

**Table 1:**
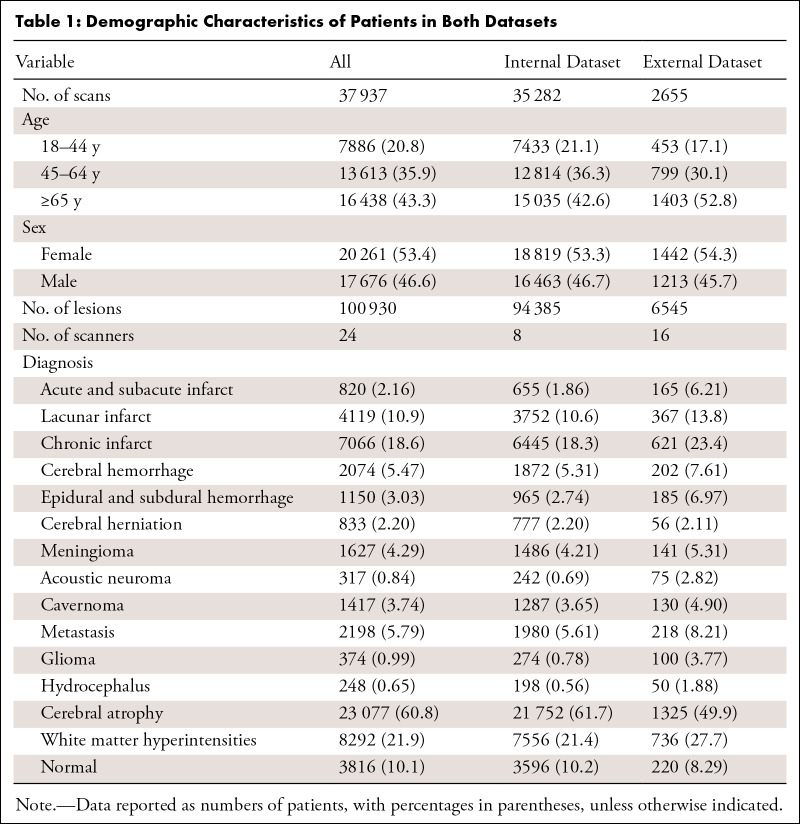
Demographic Characteristics of Patients in Both Datasets

### Radiology Report Mining Accuracy

Of the 500 randomly sampled radiologic reports, the NLP algorithm completely and correctly extracted the information of 476 (95.2%) reports. An example of the original radiology report and the corresponding structured text generated by the NLP algorithm is presented in Table S4.

### Model Diagnostic Performance

In both internal and external datasets, ReportGuidedNet consistently demonstrated higher overall performance in terms of AUC compared with PlainNet (Table 2). On the internal dataset, ReportGuidedNet had a ma-AUC of 0.93 (95% CI: 0.91, 0.95) and a mi-AUC of 0.93 (95% CI: 0.90, 0.95), surpassing PlainNet’s ma-AUC of 0.85 (95% CI: 0.81, 0.88) and mi-AUC of 0.89 (95% CI: 0.83, 0.92) (Δma-AUC, 0.08 [95% CI: 0.05, 0.11]; Δmi-AUC, −0.04 [95% CI: −0.02, 0.09]). Similarly, on the external dataset, ReportGuidedNet consistently outperformed PlainNet, with higher mi-AUC and ma-AUC values (Δma-AUC, 0.15 [95% CI: 0.12, 0.17]; Δmi-AUC, 0.15 [95% CI: 0.10, 0.18]). Specifically, as shown in Table 3, ReportGuidedNet had higher AUCs than PlainNet for all diagnoses in the internal test set (*P* = .03 to <.001). In the external test set, although no evidence of a difference was found between the two models in diagnosing hydrocephalus (*P* = .27), for the other 14 diagnoses, ReportGuidedNet had higher AUCs compared with PlainNet (*P* = .002 to <.001).

**Table 2:**
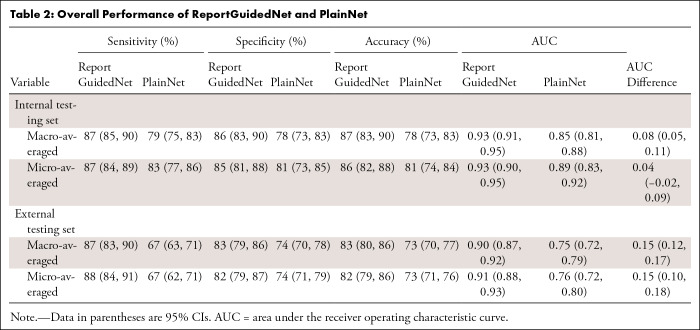
Overall Performance of ReportGuidedNet and PlainNet

**Table 3:**
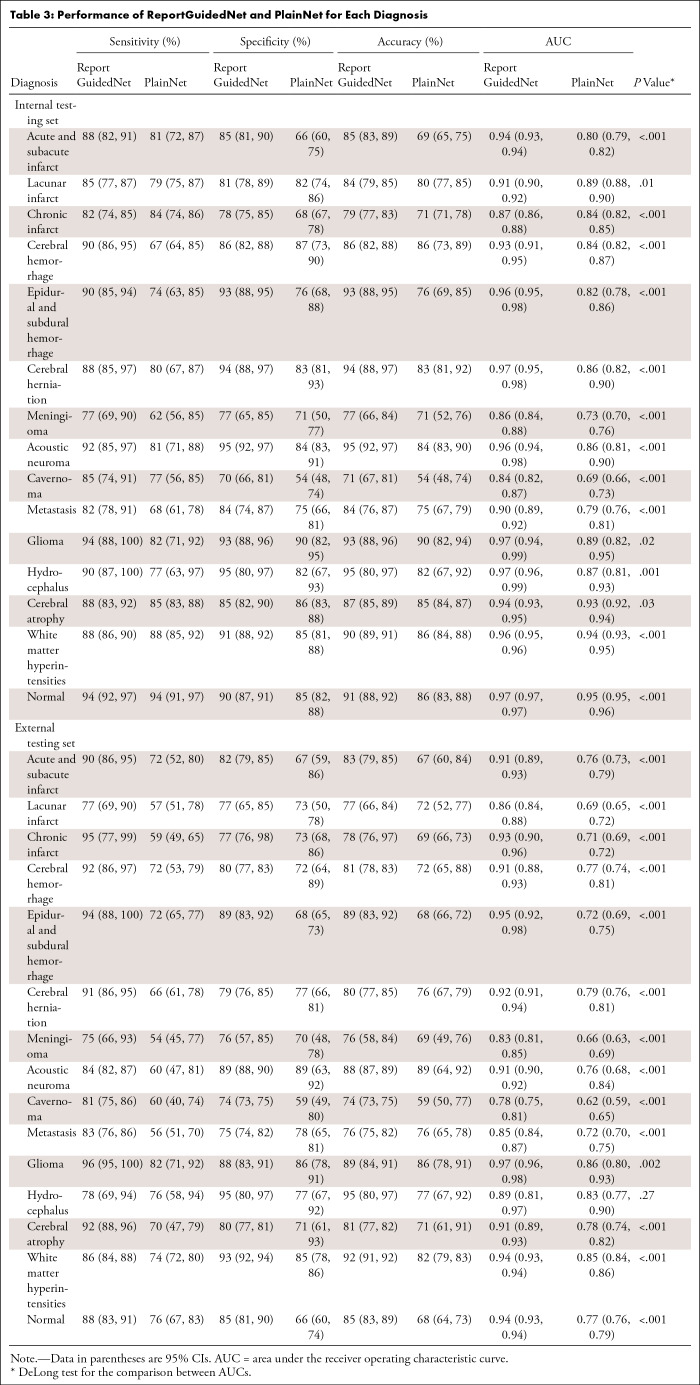
Performance of ReportGuidedNet and PlainNet for Each Diagnosis

**Figure 3:**
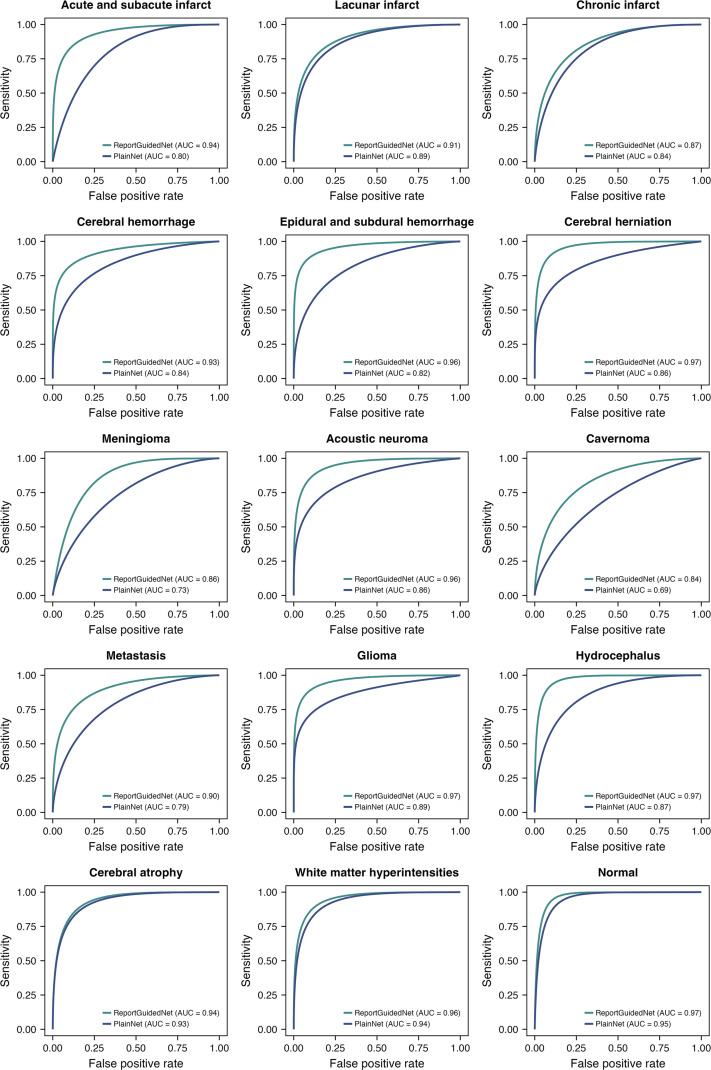
Receiver operating characteristic curves of ReportGuidedNet and PlainNet for each diagnosis. AUC = area under the receiver operating characteristic curve.

The receiver operating characteristic curves for each diagnosis for the ReportGuidedNet and PlainNet are presented in Figure 3. Calibration curves for the ReportGuidedNet are presented in section 10 of Appendix S1.

Notably, when model performance was compared on internal and external testing sets, ReportGuidedNet exhibited smaller disparities in both ma-AUC and mi-AUC than PlainNet (Δma-AUC, −0.03 vs −0.10; Δmi-AUC, −0.02 vs −0.13).

Diagnostic performance of both models on various lesion number scenarios is presented in section 2 of Appendix S1. The ma-AUC and ma-AUC of the unstructured ReportGuidedNet are 0.88 (95% CI: 0.86, 0.90) and 0.89 (95% CI: 0.85, 0.91), respectively. Detailed information can be found in section 6 of Appendix S1.

### Anomaly Localization

**Figure 4:**
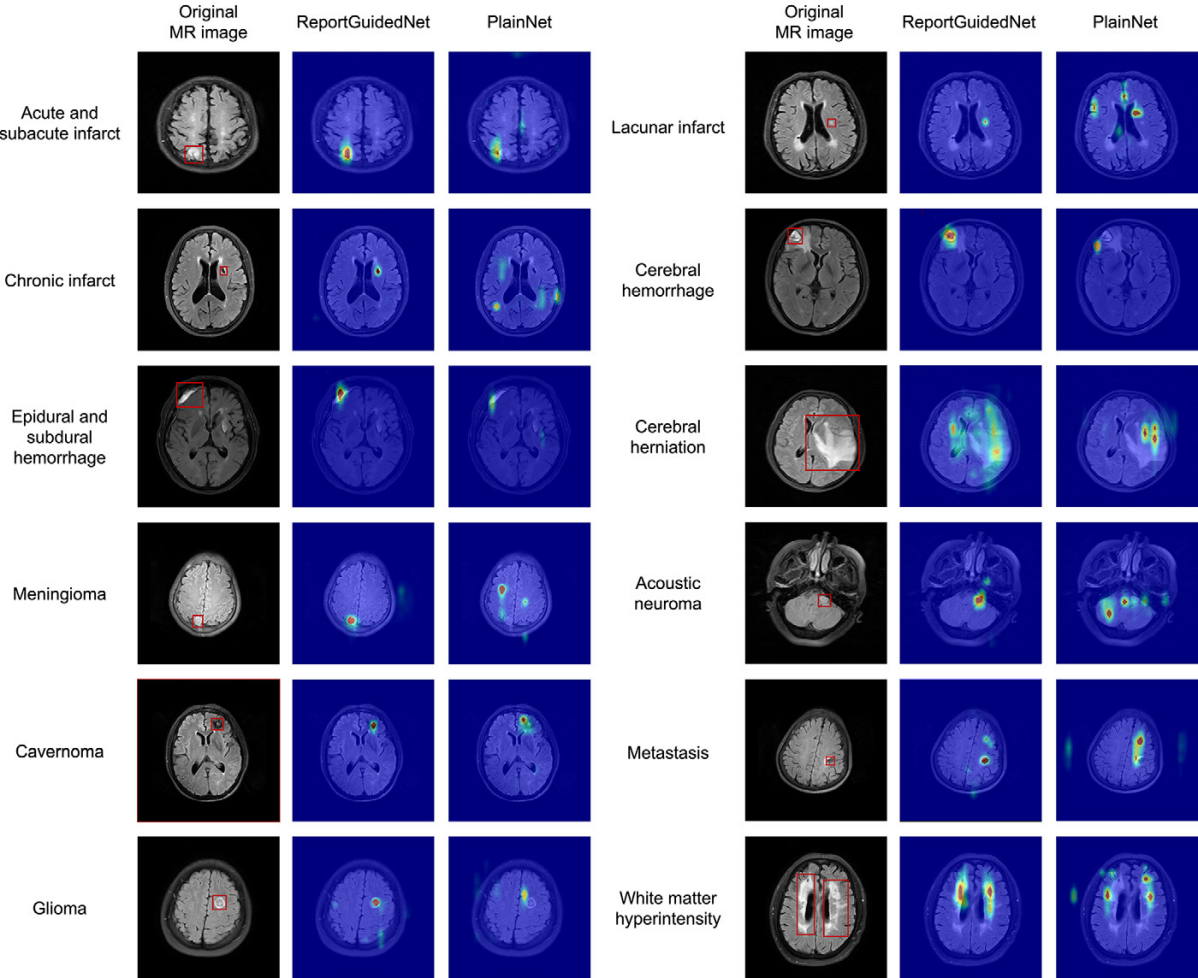
Attention maps of ReportGuidedNet and PlainNet for each diagnosis. Attention maps were not generated for cerebral atrophy, hydrocephalus, and normal brain because these conditions are diagnosed on the basis of the entire brain rather than specific regions.

**Figure 5:**
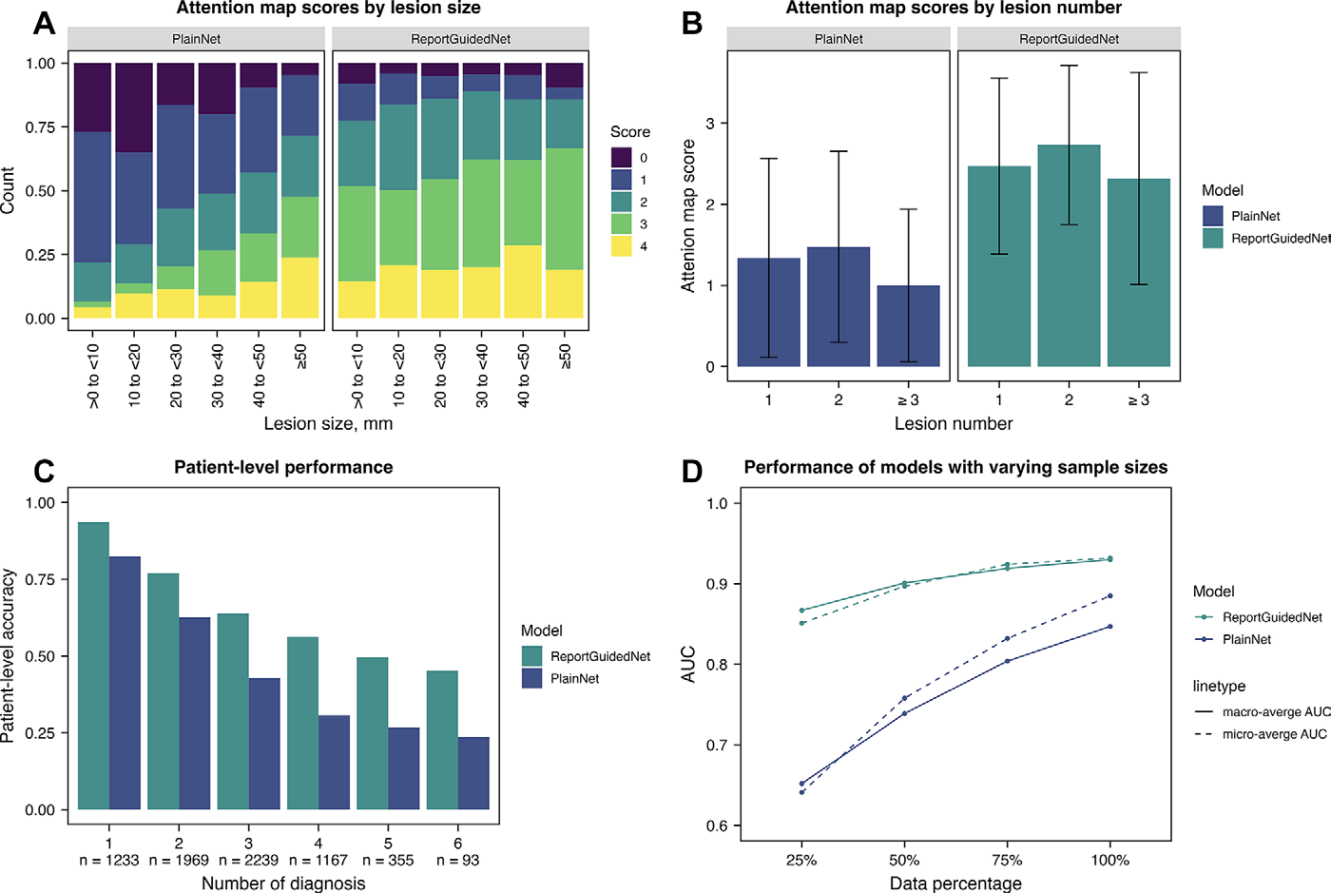
Comparison between ReportGuidedNet and PlainNet. **(A)** Attention map scores by lesion size, **(B)** attention map scores by lesion number, **(C)** patient-level accuracy of ReportGuidedNet, and **(D)** performance of ReportGuidedNet and PlainNet with varying sample sizes. AUC = area under the receiver operating characteristic curve. Error bars represent SDs.

Figure 4 demonstrates a representative attention map for each diagnosis. The Likert scale score of ReportGuidedNet (mean, 2.50 ± 1.09) was significantly higher than that of PlainNet (mean, 1.32 ± 1.20) (*P* < .001). Likert scale score of both models showed a weak but statistically significant correlation with lesion size (Fig 5A). Specifically, Likert scale score of PlainNet demonstrated relatively stronger correlation with the lesion diameter (ρ = 0.31; *P* < .001) than ReportGuidedNet (ρ = 0.09; *P* = .04). No evidence of differences was observed in the Likert scale score of ReportGuidedNet or PlainNet across scenarios involving varying numbers of lesions (Fig 5B) (ReportGuidedNet: *P* = .13; PlainNet: *P* = .12; detailed in section 8 of Appendix S1).

### Patient-level Accuracy

ReportGuidedNet demonstrated an overall patient-level accuracy of 70.3% (4958 of 7056), whereas PlainNet exhibits an overall patient-level accuracy of 52.2% (3682 of 7056). Figure 5C illustrates the accuracy of both models for patients with varying numbers of diseases.

### Data Reduction and Ablation Experiments

Figure 5D depicts the effect of training data size on the overall performance of both models. The expansion of the training data volume resulted in a gradual increase in ma-AUC for ReportGuidedNet, increasing by 0.06 from 0.87 (95% CI: 0.82, 0.91). Simultaneously, the mi-AUC of ReportGuidedNet increased by 0.08 from 0.85 (95% CI: 0.78, 0.86). For PlainNet, the ma-AUC experienced a notable elevation by 0.20 from 0.65 (95% CI: 0.63, 0.70), whereas the mi-AUC of PlainNet increased by 0.24 from 0.64 (95% CI: 0.61, 0.69). Ablation experiments and failure analyses are presented in sections 12 and 13 of Appendix S1.

### Clinical Utility

Table 4 highlights the enhanced diagnostic accuracy among radiologists when they used the ReportGuidedNet model versus when they were unassisted. ReportGuidedNet’s support notably boosted radiologists’ ability to identify brain disorders, with a particular improvement in sensitivity. For junior radiologists, improvements in macro-averaged values included increases of 0.04 in sensitivity, 0.01 in specificity, 0.02 in accuracy, and 0.03 in AUC. Micro-averaged values showed increases of 0.03 in sensitivity, 0.02 in specificity, 0.02 in accuracy, and 0.03 in AUC. There were also increases in performance metrics for senior radiologists. Macro-averaged values showed increases of 0.03 in sensitivity, 0.01 in specificity, 0.01 in accuracy, and 0.02 in AUC, and micro-averaged values showed increases of 0.02 in sensitivity, 0.01 in specificity, 0.02 in accuracy, and 0.01 in AUC. The diagnostic performance of model-assisted and unassisted junior and senior radiologists is provided in detail in section 9 of Appendix S1.

**Table 4:**
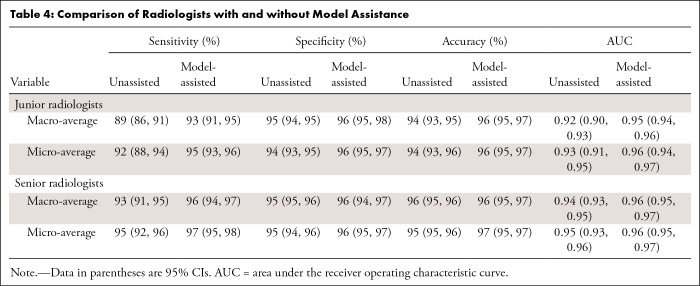
Comparison of Radiologists with and without Model Assistance

## Discussion

In this study, we harnessed textual features extracted from radiology reports, obviating the need for manual image segmentation as an annotation process, and formulated a DL model, ReportGuidedNet, capable of diagnosing multiple diseases and furnishing interpretable attention maps. ReportGuidedNet achieved higher overall diagnostic accuracy (ma-AUC, 0.93 vs 0.85; mi-AUC, 0.93 vs 0.89), with higher results across all 15 diagnoses on the internal test set (*P* = .03 to <.001) and 14 diagnoses on the external set (*P* = .002 to <.001). ReportGuidedNet also demonstrated enhanced generalizability (Δma-AUC, −0.03 vs −0.10; Δmi-AUC, −0.02 vs −0.13), more accurate localization of abnormalities (attention map score, 2.50 ± 1.09 vs 1.32 ± 1.20; *P* < .001), and lower reliance on the training data scale than the PlainNet without textual features.

In recent developments, the field of NLP has witnessed substantial advancements in the structured analysis and mining of information from radiology reports. The advent of DL technologies has heralded new avenues for the automation of data within these reports. Specifically, a variety of NLP methods have been used to automate extraction, analysis, and annotation of radiology reports, particularly to augment the extraction and organization of clinical data (15–17). The promising developments in NLP lay a solid groundwork for the training strategy proposed in this study, which uses a text feature–guided model in radiology reports to concentrate on salient features in medical imaging.

Manual image annotation, although accurate, is labor-intensive, limiting the scale of previous studies and the generalizability of models. In contrast, text derived from radiology reports provides a cost-efficient means for annotating a vast array of MR images (8,10,11), offering insights beyond localized lesions to include multiple brain anomalies. This approach not only enhances the use of medical-image data but also, as Bosma et al (18) demonstrated with prostate cancer reports, can guide semisupervised models to achieve comparable performance to that of fully supervised counterparts. Our model further refines this by focusing on key lesion characteristics informed by radiologic expertise, thereby improving disease differentiation and model interpretability (19).

The class activation map technique enhances model interpretability, highlighting crucial image regions for classification (14). ReportGuidedNet outperformed PlainNet in anomaly localization, unaffected by lesion size. This suggests that the efficiency of ReportGuidedNet in learning lesion characteristics from radiology reports enables precise identification of variously sized lesions, demonstrating better target-detection capabilities and underscoring the importance of understanding target image features for improved accuracy (20,21).

Despite the variations in imaging settings and disease prevalence across different centers, ReportGuidedNet exhibited a smaller decline in diagnostic accuracy performance than PlainNet. Thus, ReportGuidedNet showed greater generalizability. This can also be attributed to the fact that the knowledge-driven approach directed the model to prioritize lesion characteristics rather than being influenced by confounding factors, such as image parameters or normal anatomic variants.

The integration of information derived from radiology reports also enhanced learning efficiency and thereby reduced the reliance of the model on the quantity of training data. Acquisition of adequate training data remains a consistent challenge in constructing DL models, particularly in the medical imaging domain. Our findings indicate that integrating data from low-cost radiology reports offers a promising strategy to mitigate data-volume requirements.

Our analysis revealed that ReportGuidedNet significantly outperformed unstructured ReportGuidedNet in overall diagnostic performance. This can be attributed to the structured radiology reports it uses, which offer clear, concise, and targeted information, reducing ambiguity and noise. This enhances model learning efficiency and specificity. In contrast, the unstructured version of ReportGuidedNet faces challenges due to irrelevant information, inconsistent terminology, and stylistic differences in the raw texts, which hinder its ability to effectively extract and learn significant features related to disease conditions. The comparative analysis between the unstructured ReportGuidedNet and PlainNet provides a nuanced view of the utility of unstructured text data. Despite its inherent inconsistencies and complexities, unstructured text offers valuable contextual guidance that enhances overall performance, though its effectiveness varies across different diagnostic categories.

Using model diagnosis and interpretable attention maps improved radiologists’ diagnostic performance compared with the unassisted group. The attention maps aided radiologists in assessing the model’s diagnosis importance and accurately locating lesions, reducing missed diagnoses (22,23). This approach is especially valuable in regions with a specialist shortage.

Our study had several limitations. First, the range of diseases investigated was constrained by database limitations, underscoring the need for future studies to include a broader spectrum of conditions for more comprehensive diagnoses. Second, because of variations in MRI scanning protocols, we used only axial images, which may omit information from other planes but enhances model simplicity and adaptability. Third, the absence of detailed annotations limited our model’s ability to provide precise pixel-level lesion segmentation, highlighting the potential of future research to employ cost-effective annotation methods for improved accuracy. Fourth, our focus on disease-level diagnosis may not accurately reflect lesion-level localization in diseases characterized by multiple lesions, indicating a gap in our model’s ability to pinpoint lesion locations accurately in such cases. Fifth, the use of simplistic NLP techniques for the analysis of radiology reports might not fully address the variations in reporting standards, indicating a need for the investigation of more sophisticated NLP methods, such as large language models, to enhance text analysis. Last, the study’s reliance on data from tertiary care centers, which typically have fewer normal cases, points to the importance of testing the model across more varied clinical settings.

Our study integrates MRI features from radiology reports as weak annotations into our DL model. The model, which represents a cost-effective strategy that harnesses expert knowledge, showed enhanced diagnostic performance and accuracy across brain diseases compared with a model that did not use radiology report-derived textual features. This collaborative multimodal approach not only boosts diagnostic performance but also offers interpretable insights to radiologists, paving the way for advanced, efficient disease diagnosis and improved clinical decision-making.
